# Concomitant Cryoablation for Atrial Fibrillation: FREEZE-AFIB Post-Market Study

**DOI:** 10.1016/j.atssr.2025.08.011

**Published:** 2025-09-02

**Authors:** Evelio Rodriguez, Arnar Geirsson, Sabet Hashim, Patrick McCarthy

**Affiliations:** 1 Division of Cardiac Surgery, Department of Cardiac Sciences, Ascension Saint Thomas West Hospital, Nashville, Tennessee; 2 Division of Cardiology, Department of Medicine, New York-Presbyterian Columbia University Irving Medical Center, New York, New York; 3 Department of Cardiac Surgery, Harford HealthCare, Hartford, Connecticut; 4 Division of Cardiac Surgery, Department of Surgery, Northwestern University Feinberg School of Medicine and Northwestern Medicine, Chicago, Illinois

## Abstract

**Background:**

Surgical ablation is the gold standard for atrial fibrillation treatment. Cryothermal-only surgical ablation is used in clinical practice with an increasing body of published outcomes.

**Methods:**

FREEZE-AFIB (NCT05089877) was a post-market, multicenter, retrospective-prospective study to evaluate performance and safety of a commercially available cryoablation probe (cryoICE cryoFORM; AtriCure Inc.) to treat patients with nonparoxysmal atrial fibrillation. Consented patients ≥18 years who received concomitant Cox-Maze III lesions using the cryoICE cryoFORM device during cardiac surgery and returned for a prospective 12-month follow-up visit were included. Primary performance was freedom from any atrial arrhythmia >30 seconds at last follow-up without class I/III antiarrhythmic drugs. Primary safety was freedom from protocol-defined surgical ablation procedure-related serious adverse events (SAEs) (death, stroke, myocardial infarction, and major bleeding) within 30 days.

**Results:**

Thirty-three patients from 4 US sites had a mean age of 68.7 years, mean body mass index of 27.5 kg/m^2^, 76% were male, and 94% were White. Primary safety was achieved, with 100% (33 of 33) free from predefined SAEs within 30 days (95% CI, 89.42%-100%; *P* < .0001). Ninety-seven percent of patients (32 of 33) were free from SAEs and permanent pacemaker implantation through 12 months (95% CI, 84.24%-99.92%; *P* < .0001). Of 28 patients with evaluable 24-hour Holter data at 12 months, 89% (25 of 28) of patients were free from atrial arrhythmias off antiarrhythmic drugs (95% CI, 71.77%-97.73%; *P* = .001), meeting primary performance.

**Conclusions:**

Cryothermal-only surgical ablation with cryoICE cryoFORM was safe and effective, with 89% of patients free from atrial arrhythmias at 12 months off antiarrhythmic drugs and a low SAE rate.

## Visual Abstract

**Figure:**
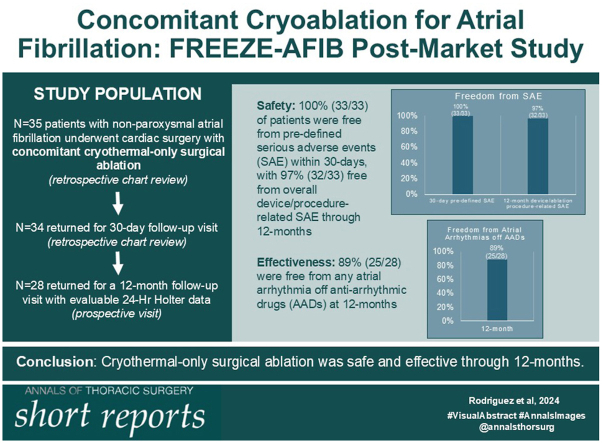

**In Short**

▪ Cryothermal-only surgical ablation was safe and effective meeting primary performance and safety endpoints.
▪ Freedom from any atrial arrhythmia off antiarrhythmic drugs at 12 months was 89% and no primary serious adverse events occurred within 30 days, with 97% of patients free from ablation procedure- and device-related serious adverse events through 12 months.
▪ Effectiveness at 12 months was higher than the 60% performance goal for the FDA trial for radiofrequency ablation clamps.

Surgical ablation (SA) is the gold standard for atrial fibrillation (AF) treatment. Current clinical practice guidelines have given class I recommendations for SA to treat AF during cardiac surgical procedures to restore sinus rhythm and improve long-term outcomes.^1^ SA can be accomplished via the Cox-Maze IV procedure using radiofrequency ablation augmented by cryothermal ablation or the Cox-Maze III procedure with cryothermal ablation alone, with excellent long-term safety and efficacy.^1^

Cryothermal energy creates cryolesions due to direct cellular injury from intracellular and extracellular ice formation during the freezing phase and microvascular stasis and subsequent ischemic necrosis during thawing.^2^ Cryothermal ablation is advantageous because it allows for visual confirmation of transmurality by formation of the “ice ball,” rapid creation of focal lesion resulting in shorter operative time, and low risk of injury to adjacent tissues due to preservation of underlying tissue architecture.^2^ Additionally, the Cox-Maze III procedure is cost-effective, requiring only 1 disposable cryoablation device for lesion set completion.^3^

We performed a post-market, retrospective-prospective study to evaluate the long-term performance and safety of concomitant cryothermal-only SA during cardiac surgery in patients with non-paroxysmal AF.

## Material and Methods

### Study Overview and Ethics Oversight

FREEZE-AFIB (NCT05089877) was a retrospective-prospective, multicenter, nonrandomized, unblinded, post-market study to evaluate long-term safety and performance of the cryoICE cryoablation system and cryoFORM cryoablation probe (CRYOF) (AtriCure Inc.) to treat persistent and long-standing persistent AF. The CRYOF US indication is the cryosurgical treatment of cardiac arrhythmias by freezing target tissues, creating an inflammatory response that blocks electrical conduction. The study was sponsored/funded by AtriCure, Inc.

The study was conducted in accordance with the Declaration of Helsinki, applicable Good Clinical Practices and regulations (US 21 CFR Part 50, 21 CRF Part 56, 21 CFR Part 812, and BS EN ISO14155:2020). Prior to enrollment, the study was approved by the institutional review board for each investigational site (Supplemental Table 1).

### Patient Eligibility

Patients ≥18 years with documented history of AF who underwent cardiac surgery with concomitant SA for AF using CRYOF were eligible for inclusion. The minimum lesion set was based on the left atrial Cox-Maze III lesions. Full inclusion/exclusion criteria are included in Supplemental Table 2. To mitigate temporal bias, a deidentified list of potential study patients was randomized by the sponsor and provided to the site for screening. Patients provided informed consent.

### Study Visits and Follow-Up

The study design included retrospective and prospective data collection per the study visit schedule (Supplemental Table 3).

### Primary and Secondary Endpoints

The primary performance endpoint was freedom from any documented AF, atrial flutter (AFL), or atrial tachycardia (AT) >30 seconds at last follow-up visit without class I/III antiarrhythmic drugs (AADs) (except for previously failed AADs at doses not exceeding those previously failed). Subsequent catheter ablation or SA treatment for AF/AFL/AT after the index SA procedure was also considered a failure. There was no blanking period postprocedure where episodes were treated but not counted against success. Twenty-four-hour Holter monitoring data were evaluated by the independent core laboratory using a standardized evaluation protocol. The performance goal (PG) for primary performance was 55%.

The composite primary safety endpoint was the incidence of the following protocol-defined serious adverse events (SAEs) within 30 days of the ablation procedure, if related to the SA procedure: death, stroke (regardless of level of disability), myocardial infarction, and major bleeding events. Deaths beyond 30 days and through last follow-up attributed to the SA procedure or CRYOF device were reported as SAEs. All SAEs were adjudicated by an independent physician from a contract research organization (Avania, LLC). The PG for safety was 15%.

Secondary performance endpoints included (1) freedom from any documented AF/AFL/AT lasting >30 seconds at last follow-up visit, regardless of AADs, (2) freedom from any documented AF/AFL/AT >30 seconds at last follow-up visit without class I/III AADs, and (3) acute procedural success defined as documentation of sinus rhythm at the end of the procedure. Secondary safety endpoints included (1) freedom from permanent pacemaker (PPM) implantation postprocedure through last follow-up and (2) freedom from overall device- or SA procedure-related SAEs through last follow-up.

### Sample Size Calculation and Statistical Analyses

Sample size calculation and statistical methods are presented in the Supplemental Methods.

## Results

**Figure 1:**
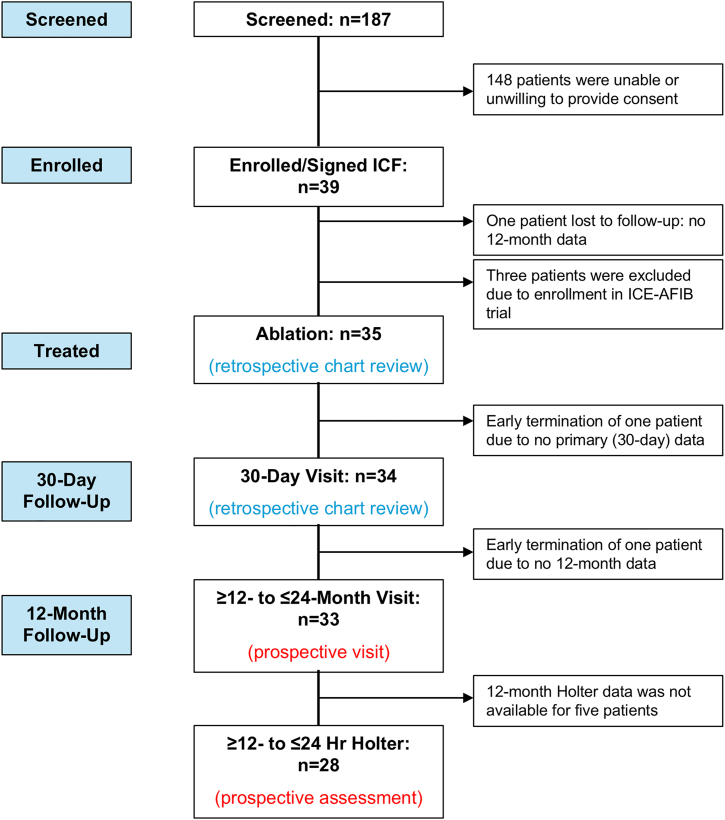
Disposition of participants. (ICF, informed consent form.)

Thirty-nine patients consented and were enrolled from 4 investigational sites in the U.S. from April 28, 2022, to March 21, 2023 (Figure 1). In the intent-to-treat population, the mean age was 68.7 years, mean body mass index was 27.5 kg/m^2^, 76% were male, and 94% were White (Table 1). Most patients had persistent AF (85%; 28 of 33), while 15% (5 of 33) had longstanding persistent AF. Medical history is shown in Supplemental Table 4. Concomitant operations performed included mitral valve (81.8%; 27 of 33), tricuspid valve (21.2%; 7 of 33), coronary artery bypass graft (12.1%; 4 of 33), aortic valve (12.1%; 4 of 33), or other cardiac surgeries (18.2%; 6 of 33) (Table 2).

**Table 1:** Baseline Clinical Characteristics and Demographics

Characteristic	Result
Age, y	(N = 33)
Mean	68.7
Median	69
Range	53, 83
Sex	(N = 33)
Male	75.8 (25)
Female	24.2 (8)
Race	(N = 33)
White	93.9 (31)
Black	3.0 (1)
Other	3.0 (1)
Ethnicity	(N = 33)
Not Hispanic/Latino	97.0 (32)
Hispanic/Latino	3.0 (1)
Body mass index, kg/m^2^	(N = 30)
Mean	27.5
Median	26.15
Range	20.2, 44.8
Continuous AF at baseline	(N = 33)
Yes	100 (33)
No	16.8 (26)
Prior antiarrhythmic drug failure	(N = 33)
Yes	3.0 (1)
No	97.0 (32)
Atrial arrhythmia	(N = 33)
Persistent AF	85.0 (28)
Longstanding Persistent AF	15.0 (5)
Left atrium diameter, cm	(N = 29)
Mean	4.7
Median	4.7
Range	3.3, 6.7
Left ventricular ejection fraction, %	(N = 33)
Mean	57.9
Median	58
Range	37, 75
New York Heart Association classification	(N = 33)
I	12.1 (4)
II	39.4 (13)
III	12.1 (4)
IV	0.0 (0)
No heart failure	3.0 (1)
Not assessed^a^	33.3 (11)

Values are presented as % (n), unless otherwise noted.

AF, atrial fibrillation.

a Not reported/not assessed were selection choices on the case report form and do not indicate missing data.

**Table 2:** Procedural Data

Procedural Data	% (n)
**Concomitant Surgical Procedure**^a^**(N = 33)**	
Mitral valve	81.8 (27)
Tricuspid valve	21.2 (7)
Coronary artery bypass graft	12.1 (4)
Aortic valve	12.1 (4)
Other	18.2 (6)
LAA MAZE	3.0 (1)
MAZE, LAA closure	3.0 (1)
Tricuspid valve annuloplasty, LA MAZE, LAA closure	3.0 (1)
Vascular graft	3.0 (1)
**Lesion Sets (N = 33)**	**% (n)**
Right atrial endocardial lesions	87.9 (29)
Left atrial endocardial and epicardial (CS) lesions	100.0 (33)
**Left Atrial Appendage Management (N = 33)**	**% (n)**
Left atrial appendage excluded	93.9 (31)
AtriClip	66.7 (22)
Suture	24.2 (8)
Cut-and-sew	3.0 (1)
LAA not managed^b^	6.1 (2)

CS, coronary sinus; LA, left atrium; LAA, left atrial appendage.

a Multiple procedures could be performed; primary reason for surgery was not collected.

b Prior occlusion of LAA suggested in comments: (1) Watchman and (1) left atriotomy noted as completed (did not state LAA).

Of 28 patients who had evaluable 24-hour Holter monitor data, primary performance was achieved with 89.3% (25 of 28) free from AF/AFL/AT off class I/III AADs except AADs at doses not exceeding those previously failed (*P* = .0001 compared with PG). No new/increased AADs at 12 months or repeat ablation procedures were reported.

Of 33 patients in the intent-to-treat population, no primary SAEs were adjudicated as related to the device or SA procedure; thus, 100% (95% CI, 89.42%-100%) of patients achieved primary safety success (*P* < .0001 compared with PG).

For secondary performance endpoints, 89.3% (25 of 28; 95% CI, 71.77%-97.73%; *P* = .0001) of patients achieved freedom from AF/AFL/AT at 12 months regardless of AADs. Procedural success (sinus rhythm at end of ablation procedure) was 71.4% (20 of 28; 95% CI, 51.33%-86.78%; *P* = .0578).

One patient failed both secondary safety endpoints due to a SAE related to the SA procedure requiring PPM implantation within 30 days of ablation procedure. Thus, 96.97% (95% CI, 84.24%-99.92%; *P* < .0001) of patients were free from SAEs related to the SA procedure and PPM implantation through 12 months postablation procedure. One patient (3%; 1 of 33) experienced an acute kidney injury, adjudicated as unrelated to the device or ablation procedure. There were no accounts of direct current cardioversion within 12 months postprocedure.

## Comment

Cryothermal-only SA with the CRYOF device achieved primary performance, with 89.3% of patients free from atrial arrhythmias through 12 months, without new/increased class I/III AADs. This is comparable to 12-month efficacy rates in published literature for SA during concomitant cardiac procedures.^1^ Furthermore, the efficacy rate in FREEZE-AFIB is comparable to a recent study by McCarthy and associates^3^ that reported 90% of patients who underwent concomitant cryothermal-only SA during mitral valve surgery experienced freedom from AF off AADs at 12 months. Two prospective, multicenter trials of the Cox-Maze IV procedure using radiofrequency clamp ablation systems reported freedom from AF off AADs for 53% of patients between 6 and 9 months (CURE-AF [Concomitant Utilization of Radiofrequency Energy for Atrial Fibrillation] trial) and 76% at 6-months (ABLATE [AtriCure Bipolar Radiofrequency Ablation of Permanent Atrial Fibrillation] trial).^4^^,^^5^

The primary safety endpoint was met with 100% of patients free from ablation procedure-related SAEs within 30-days. Ninety-seven percent of patients were free from SAEs related to the SA procedure and PPM implantation through 12 months. Several studies have reported that SA is associated with an increased risk of perioperative PPM implantation and new renal dysfunction.^1^^,^^6^ In FREEZE-AFIB, 1 patient (3%) underwent concomitant SA during mitral valve repair and experienced sick sinus syndrome leading to a complete heart block 26 days post-index procedure that required implantation of a PPM. The 3% PPM implantation rate is in the range reported for patients undergoing cardiac surgery without SA from the STS database.^6^ While new renal dysfunction was not a specific safety endpoint of FREEZE-AFIB, adverse event reporting identified 1 acute kidney injury, which was adjudicated as unrelated to the device or ablation procedure.

This study is limited by the retrospective component of the trial design, which may have introduced selection bias, as well as low trial enrollment and small sample size. Additionally, arrhythmias are reported at last follow-up visit rather than through last follow-up, so it is possible that episodes occurred within the study period but were not detected.

In conclusion, cryothermal-only SA ablation during concomitant cardiac operations was safe and effective. With 89% free from AF/AFL/AT through 12 months off AADs, cryothermal-only SA achieved higher efficacy than previously reported for radiofrequency clamps for freedom from AF/AFL/AT.

## References

[1] Wyler von Ballmoos M.C., Hui D.S., Mehaffey J.H. (2024). The Society of Thoracic Surgeons 2023 clinical practice guidelines for the surgical treatment of atrial fibrillation. Ann Thorac Surg.

[2] Cox J.L., Malaisrie S.C., Churyla A. (Aug 2021). Cryosurgery for atrial fibrillation: physiologic basis for creating optimal cryolesions. Ann Thorac Surg.

[3] McCarthy P.M., Cox J.L., Kruse J., Elenbaas C., Andrei A.C. (2024). One hundred percent utilization of a modified CryoMaze III procedure for atrial fibrillation with mitral surgery. J Thorac Cardiovasc Surg.

[4] Damiano R.J., Badhwar V., Acker M.A. (2014). The CURE-AF trial: a prospective, multicenter trial of irrigated radiofrequency ablation for the treatment of persistent atrial fibrillation during concomitant cardiac surgery. Heart Rhythm.

[5] Philpott J.M., Zemlin C.W., Cox J.L. (2015). The ABLATE trial: safety and efficacy of Cox Maze-IV using a bipolar radiofrequency ablation system. Ann Thorac Surg.

[6] Badhwar V., Rankin J.S., Ad N. (2017). Surgical ablation of atrial fibrillation in the United States: trends and propensity matched outcomes. Ann Thorac Surg.

