# Supplementary material for: Concomitant Cryoablation for Atrial Fibrillation: FREEZE-AFIB Post-Market Study
Source: Ann Thorac Surg Short Rep. 2025 Sep 2;4(1):318–23. doi: 10.1016/j.atssr.2025.08.011 (PMC13100763; doi:10.1016/j.atssr.2025.08.011)
Supplement: Supplementary Material [file mmc1.docx]

**Supplemental Material**

**Supplemental Methods**

*Sample Size Calculation*

Based on a PG of 55% success and an expected success rate of 68%, a sample size of 140 patients would have provided >88% power using a one-sided Exact test, with α = 0.025 significance to demonstrate primary performance success. Thus, target enrollment was up to 150 patients, assuming an attrition rate of 6.5% for last follow-up visit. Due to slow enrollment during the pandemic and despite all efforts to accelerate patient enrollment, enrollment in the FREEZE-AFIB study was stopped after 39 patients were enrolled.

Based on the above assumptions and given the actual number of patients enrolled, the updated lowest primary performance endpoint that would meet the PG was 75% (21/28 patients free from AF, AFL, or AT episode >30 seconds at last follow-up in the absence of Class I/III AADs).

Based on the primary safety performance goal of 15% and an expected population SAE rate of up to 8.5%, a sample size of 150 patients would have 80% power using a one-sided Exact test, at α = 0.025 significance. With these assumptions and given the actual number of subjects enrolled, the updated lowest primary performance endpoint that would meet the PG would be 3% (1/34 patients experience at least one primary SAE).

*Statistical Analysis*

For the primary performance endpoint of freedom from any documented AF, AFL, or AT lasting >30 seconds at last follow-up in the absence of Class I/III AADs, an exact binomial test was conducted at a one-sided α = 0.025 level of significance to test the following hypothesis that the proportion of performance successes was significantly higher than the PG:

H_0_: *p* ≤ 55%

H_A_: *p* > 55%

where *p* was the responder rate at last follow-up visit occurring at least 12-months post-procedure. The per-protocol (PP) population was the primary population for this analysis.

For the primary safety endpoint of freedom from protocol-defined SAEs, an exact binomial test was conducted at a one-sided α = 0.05 level of significance to test the following hypothesis that the proportion of patients who had SAE was significantly lower than the PG:

The hypothesis test for the safety endpoint was:

H_0_: *q* ≥ 15%

H_A_: *q* < 15%

where *q* was the proportion of patients with SAEs through 30-days of index ablation procedure. The intent-to-treat (ITT) population was the primary population for this analysis.

Standard descriptive statistics were used to summarize numeric variables, including the number of observed values, mean, standard deviation, median, minimum and maximum values. Summaries of categorical variables included the number and percentage of observed values at each level of the categorical variable.

Statistical analysis was performed by an independent contract research organization, Clintera LLC (Orange, NJ) using SAS 9.4.

**Supplemental Table 1.** Institutional Review Boards for Investigational Sites

| **Institution** | **Institutional Review Board (IRB)** | **Approval Date** | **ID #** |
| --- | --- | --- | --- |
| Hartford Healthcare | Hartford Healthcare IRB | May 4^th^, 2022 | #FWA00021932 |
| Northwestern University | Northwestern IRB | February 16^th^, 2022 | #FWA00001549 |
| Saint Thomas West Hospital | Sterling IRB | February 8^th^, 2022 | #FWA00001790 |
| Yale University School of Medicine | Yale IRB | February 4^th^, 2022 | #FWA00002571 |

**Supplemental Table 2.** Patient Selection Criteria

| **Inclusion Criteria** | **Exclusion Criteria** |
| --- | --- |
| ≥18 years of age | Stand-alone AF without indication(s) for concomitant CABG and/or valve surgery |
| Documented history of AF | Previous left-sided ablation procedures prior to surgical ablation |
| Received surgical ablation for AF using CRYOF | Untreated atrial flutter and symptomatic ventricular arrythmia |
| Lesion set included at least the following lesions, with a lesion duration of at least 2 minutes: | Known carotid artery stenosis greater than 80% prior to index ablation procedure |
| - Left and right pulmonary vein isolation | Prior history of ischemic stroke or hemorrhagic stroke |
| - Roof and floor lines | History of myocardial infarction with ST elevation within 6 weeks prior to the index ablation |
| - Mitral annulus line | Documented AF duration of greater than 10 years |
| - Connecting lesion from LAA to left pulmonary vein | Large left atrial size (i.e., LA diameter > 7 cm) prior to the index ablation procedure |
| - Coronary sinus lesion | Active systemic infection prior to index ablation procedure |
| - LAA exclusion | Documented severe peripheral arterial occlusive disease defined as claudication with minimal exertion prior to the ablation procedure |
| Subject underwent non-emergent cardiac surgical procedure(s) on cardiopulmonary bypass including open-heart surgery for one or more of the following: | History of renal failure requiring dialysis or hepatic failure prior to the ablation procedure |
| - Mitral valve repair or replacement | Known drug and/or alcohol addiction |
| - Aortic valve repair or replacement | Mental impairment or other conditions which may not allow the subject to understand the nature, significance, and scope of the study |
| - Tricuspid valve repair or replacement | Pregnancy |
| - CABG procedures | Preoperative need for mechanical circulatory support or intravenous inotropes |
| - Atrial septic defect repair | Anti-arrhthmic drug therapy for the treatment of another arrhythmia |
| LVEF ≥30% (determined by echocardiography or cardiac catheterization performed within 90 days of enrollment as documented in subject medical history) | Currently undergoing chemotherapy |
| Willing and able to provide written informed consent | Long-term treatment with oral or injected steroids (not including intermittent use of inhaled steroids for respiratory disease) |
| Willing and able to return for scheduled follow-up visits | Known connective tissue disorders at the time of index ablation procedure |
|  | Known hypertrophic obstructive cardiomyopathy at the time of index ablation procedure |
|  | Known cold agglutinin |
|  | COVID-19 infection |
|  | Bleeding disorders and/or inability to receive anticoagulation |
|  | Aortic dissection surgery as index procedure |
|  | Cardiac surgical re-intervention since the index cardiac surgery with concomitant AF ablation procedure |

AF: atrial fibrillation; CABG: coronary artery bypass graft; LAA: left atrial appendage; LVEF: left ventricular ejection fracture

**Supplemental Table 3.** Study Visit Schedule

| **Trial Activity** | **Visit 1** | **Visit 2** | **Visit 3** | **Visit 4** | **Visit 5** |
| --- | --- | --- | --- | --- | --- |
|  | **Baseline (within 30 days of index procedure)** | **Procedure** | **Pre-Discharge** | **30-Days (within 23-37 days of index procedure)** | **12-Months (≥ 12-months but ≤ 24-months of index ablation procedure)** |
| Informed Consent and Inclusion/Exclusion | X |  |  |  |  |
| Demographics | X |  |  |  |  |
| Medical/Surgical History | X |  |  |  |  |
| AF Classification | X |  |  |  |  |
| Vital History | X |  |  |  |  |
| NYHA Classification | X |  |  | X | X |
| 12-Lead ECG, if done^1^ | X | X | X | X |  |
| Medications/AAD Adjustment Review^2^ | X |  | X | X | X |
| Adverse Events (AEs/SAEs) |  | X | X | X | X |
| Echocardiogram (transthoracic), if done | X |  |  |  |  |
| Cox Maze III |  | X |  |  |  |
| Concomitant Surgical Procedure |  | X |  |  |  |
| Cardioversion Review^3^ |  | X | X | X | X |
| 24 Hour-Holter (or equivalent) |  |  |  |  | X |
| AE: adverse events; AF: atrial fibrillation; AAD: antiarrhythmic drugs; ECG: electrocardiogram; NYHA: New York Heart Association; SAE: serious adverse events  ^1^12-lead ECG performed was 30 seconds in length  ^2^Class I/III AADs and/or OACs only  ^3^At physician’s discretion and per institutions standard of care | | | | | |

**Supplemental Table 4.** Medical History

| **Characteristics** | **% (n/N)** |
| --- | --- |
| Hypertension | 45.5% (15/33) |
| Other | 42.4% (14/33) |
| Hyperlipidemia | 30.3% (10/33) |
| Coronary Heart Disease | 18.2% (6/33) |
| Sleep Apnea | 12.1% (4/33) |
| Cancer | 6.1% (2/33) |
| Congestive Heart Failure | 6.1% (2/33) |
| Diabetes Mellitus | 6.1% (2/33) |
| Cardiomyopathy | 3.0% (1/33) |
| Myocardial Infarction | 3.0% (1/33) |
| Pulmonary Hypertension | 3.0% (1/33) |
| Rheumatic Heart Disease | 3.0% (1/33) |
| Transient Ischemic Attack | 3.0% (1/33) |
